# Development of patty meat analogue using anchovy protein isolate (*Stolephorus insularis*) as a binding agent

**DOI:** 10.1016/j.heliyon.2023.e23463

**Published:** 2023-12-10

**Authors:** Meda Canti, Juan Owen, Maximilliam Febriand Putra, Rory Anthony Hutagalung, Novia Utami

**Affiliations:** a Food Technology Study Program, Faculty of Biotechnology, Atma Jaya Catholic University of Indonesia, Tangerang, 15345, Indonesia; b Master of Biotechnology Study Program, Faculty of Biotechnology, Atma Jaya Catholic University of Indonesia, Jakarta, 12930, Indonesia; c Management Study Program, Faculty of Economics and Business, Atma Jaya Catholic University of Indonesia, Jakarta, 12930, Indonesia

**Keywords:** Anchovy, Patty meat analogue, Protein isolate, Physicochemical, Sensory

## Abstract

The development of meat analogues focuses on sustainable production and requires attention to their nutritional, physicochemical, and sensory values. Anchovy protein isolate (API) is a novel and potential binding agent in the development of meat analogues. This study aimed to produce API and evaluate the physical, proximate, and sensory qualities of patty meat analogue (PMA) with the addition of API. The preparation method for API uses pH-shifting. The ratios of API added to the meat analogues were 0 % (F0), 4 % (F1), 8 % (F2), and 12 % (F3) per textured vegetable protein (TVP) weight. Furthermore, PMA was analysed for physical, proximate, and sensory properties. API had 87.23 % dry basis (db) protein content. The amino acid composition of API generally complied with the nutritional requirements of adults and children. The addition of API significantly affected the physical properties, proximate composition, and sensory (taste) qualities of PMA *(p* < 0.05). The protein content of PMA met Indonesian national standards (SNI) and was similar to both McDonald's and ground beef patty based on United States Department of Agriculture (USDA) standards. F3 was found to be the best based on its physical, proximate, and sensory properties.

## Highlights

• Innovations meat analogue with anchovy protein isolate (API) was studied.
• API was rich in protein and essential amino acids and could be used as a binder in patty meat analogues.
• Adding API increased texture and reduced the cooking loss of patty meat analogues.
• The addition of API improved the nutritional of patty meat analogue.
• Sensory evaluation showed that meat analogue with adding API was acceptable in all attributes.

## 1 Introduction

Meat is the primary source of protein for most of the world's population. The world's population is predicted to increase significantly to 9.8 billion in 2050 and 11.2 billion in 2100, causing an increase in the consumption of animal protein [1]. The animal protein industry can negatively impact the sustainability of the environment and human health. In recent years, the level of consumption of animal meat, by some groups, has begun to decrease due to the high price of beef and chicken, and there is a movement to stop the slaughter of meat-producing animals. High consumption of animal protein can increase greenhouse gas (CO_2_) emissions [2]. In addition, meat production can lead to excess use of land and water resources, and animal disease risk, harming the environment. Long-term red or processed meat consumption has been shown to cause various diseases, such as cardiovascular complications and cancer [3]. Therefore, meat substitute products are needed for consumers who want to reduce meat consumption, but still consume protein where the texture, taste, and appearance are similar to meat.

Meat analogue is a product that resembles meat in appearance, taste, texture, and nutritional content and can be free of meat or still contain a small amount of meat [4]. Meat analogue is also known as imitation meat, or meat substitute, which can be partial or full substitutes [5]. Currently, meat analogue is gaining popularity as an alternative to meat because it has many health benefits, such as less fat content than animal meat. A good meat analogue has a chewy, juicy, not brittle texture, no aroma, and no intense beany flavour [6]. Generally, meat analogues are made from vegetable ingredients such as gluten, soy, and soy-derived products such as textured vegetable protein (TVP). Research on meat analogue products has developed significantly by combining various ingredients to increase the nutritional, physicochemical, and sensory values of meat analogues. Several studies on the preparation of meat analogues have been carried out with various formulations of ingredients, namely cricket flour and soy protein isolate [7]; rapeseed protein concentrate [8]; oat fibre concentrate and pea protein isolate [9]; and pea, wheat and soy protein [10]. In manufacturing meat analogues, adding binding agents that increase water binding capacity, texture, and emulsification characteristics, such as wheat gluten, egg white, starch, protein isolate and concentrate, is necessary.

Protein isolate is a powder preparation with a protein content of up to 90 % or more of its dry weight. Protein isolate can be made from plant and animal materials. Research on making protein isolates from various types of fish have been developed. Fish protein isolates are produced from Indian mackerel, salmon, cod, herring by-products, ponyfish, sardines, lanternfish, yellowfin tuna roe, tilapia, anchovy, Baltic herring, and catfish [[11], [12], [13], [14], [15], [16], [17], [18]]. The functional protein isolate is added to food products, stabilizing the emulsion, increasing colour brightness, improving texture, increasing protein content, and reducing cooking loss [19]. In meat analogue products, protein isolate acts as a binder. Research by de Angelis et al. [20] showed that the addition of pea protein isolate and soy protein isolate to meat analogues can increase protein content, texture, water absorption capacity (WAC), and oil absorption capacity (OAC). In addition, Yuan et al. [21] research also showed that analogue sausages based on edible mushrooms and soy protein isolate had a texture profile similar to real beef.

The raw material for preparing protein isolates in this study used anchovies. Anchovies are one of Indonesia's most common types of seawater fish, with catches in 2021 of 172,818.74 quintals [22]. The protein content of anchovies is also high; in fresh fish, the content is 16.32–22.20 %, while in dried fish, it is 60.20–68.82 % [23]. Based on the Indonesian Statistics Agency [24], the level of consumption of anchovies in Indonesia is 0.008 kg/capita a week. In addition, the growth of anchovies is not affected by the seasons so they have the potential for development. One of the most common anchovies found is jengki anchovy (*Stolephorus insularis*). The use of anchovies in Indonesia is still limited to namely, dried, salted, and fried, such as salted anchovies, dried anchovies, *orek* anchovies, and anchovy chips. Therefore anchovies have the potential to be developed into protein isolates. Anchovy protein isolate (API) can improve the emulsion stability and protein content in processed formulations of meat products [14]. No studies have used TVP and API to manufacture patty meat analogue (PMA). This study aimed to produce API and evaluate the physical, sensory, and proximate PMA quality by adding API.

## 2 Materials and methods

### 2.1 Materials

The material used in this study was fresh anchovy (FA) (*Stolephorus insularis*) obtained from Kampung Bebek Market, West Jakarta, Indonesia. Textured vegetable protein (TVP) was purchased from Multi Chemical Indotrading (Bandung, Indonesia), where the TVP originated from India. TVP had 7.63 % moisture, 6.38 % ash, 52.36 % protein, 0.42 % lipid, 3.45 % crude fibre, and 33.21 % carbohydrate content. Other ingredients used were red yeast rice powder (Jaya Bakti Sembako), shallot powder (Jay's Kitchen), black pepper (Jay's Kitchen), oregano (Jay's Kitchen), tomato sauce (Del Monte), chilli powder (Jay's Kitchen), sago flour (Pak Tani), tomatoes, and salt. Absolute ethanol was obtained from PT Nusa Kimia (Jakarta, Indonesia). 6-Aminoquinolyl-N-hydroxysccinimidyl carbamate (AQC) was acquired from MedChemExpress (PT Genetika Science Indonesia). Kjeltabs protein was obtained from Behr Labor-Technik GmbH (PT Besha Analitika, Jakarta, Indonesia). All other analytical chemical reagents were purchased from Sigma-Aldrich (PT Merck Chemicals and Life Sciences, Jakarta, Indonesia).

### 2.2 Preparation of anchovy flour (AF)

The anchovy flour (AF) preparation follows Canti et al. [14]. First, 5 kg of fresh anchovies (FA) were washed and then ground into anchovy pulp using a food processor (Mitochiba CH-100, Indonesia). Next, the anchovy pulp was dried in a cabinet dryer (PT Agrowindo Sukses Abadi OVL-12, Indonesia) at 55 °C for 22 h. The anchovy flour was then pureed, again with a food processor, to get a smooth texture and sieved using an 80-mesh sieve (Megah Gumilang Chemikatama ATE-210, Indonesia). The moisture content of the anchovy flour obtained was 6.26 % db.

### 2.3 Preparation of defatted anchovy flour (DAF)

The preparation of defatted anchovy flour (DAF) followed the method of Canti et al. [14]. The lipid content of anchovy flour was removed using 96 % food-grade ethanol with a 1:3 (w/v) ratio. The extraction process used a hotplate stirrer (Thermo Scientific HPS RT2 Basic, USA) for 30 min to ensure the AF was homogeneous. After that, the mixture was centrifuged (Eppendorf 5810, Germany) for 20 min at a speed of 1,811×*g*. Pellets resulting from centrifugation were defatted anchovy flour. This stage was carried out three times. Finally, the defatted flour was stored overnight in a fume hood (Biobase FH1000, China) to evaporate the remaining ethanol. The moisture content of defatted anchovy flour obtained was 11.74 % db.

### 2.4 Preparation of anchovy protein isolate (API)

The preparation of API followed the method of Canti et al. [14] with a slight modification of centrifugation speed. Defatted flour was extracted at pH 11 using 0.1 N NaOH at a ratio of 1:20 for 30 min. The pH was checked twice every 15 min to ensure the pH remained at 11. Detention at pH 11 was based on the pH shifting method because pH 11 was the condition where the protein had the highest solubility. After that, the mixture was centrifuged at 2,465×*g* for 30 min. The supernatant from centrifugation would precipitate at pH 5 with 0.1 N HCl. The degree of acidity of five was the isoelectric point. This step was followed by centrifugation at 2,465×*g* for 30 min. The supernatant was separated, and the pellets obtained were dried in a freeze dryer (Christ Alpha 2–4 LDPlus, Germany) at −35 °C for 96 h.

### 2.5 Preparation of patty meat analogue (PMA)

The preparation of PMA was carried out based on the method of Bakhsh et al. [25]. In the meat analogue formulation, there were four treatments with different concentrations of API per weight of TVP used. The treatments were F0 (0 %), F1 (4 %), F2 (8 %), and F3 (12 %). For the preparation of tomato juice, the tomatoes were squeezed and filtered to produce tomato juice. After that, 6 g of red yeast rice powder, 5 g of shallot powder, 12 g of tomato sauce, 2 g of oregano, 2 g of chilli powder, 1.5 g of salt, and 1.5 g of black pepper were added to 220 mL of tomato juice. Then, the tomato juice was boiled at 80 °C for 15 min until all the spices were dissolved. The tomato juice was then cooled to room temperature (25 °C). Afterwards, the tomato juice was mixed with API according to the treatment, 100 g of TVP, and 20 g of sago flour. Then the meat analogue dough was moulded using a patty mould with a diameter of 6 cm. The dough was steamed at 100 °C for 15 min and stored in a freezer (GEA AB-600TX, Indonesia) at −20 °C. Finally, before consuming the meat analogue, it was first fried at 175 °C for 2 min.

### 2.6 Yield and protein recovery of anchovy protein isolate (API)

The yield and protein recovery of API from FA was calculated using equations (1), (2), respectively [13].

(1) $$Yield\;of\;API\;\left(\%\;wb\right)=\frac{weight\;of\;anchovy\;protein\;isolate\;\left(API\right)\;\left(g\right)}{\;weight\;of\;fresh\;anchovy\;\left(FA\right)\;\left(g\right)}x\;100\%$$

(2) $$Protein\;Recovery\;of\;API\;\left(\%\;wb\right)=\frac{protein\;content\;in\;API\;\left(\%\;db\right)\times weight\;of\;API\;\left(g\right)}{protein\;content\;in\;FA\;\left(\%\;db\right)\times weight\;of\;FA\;\left(g\right)}\times 100\%$$

### 2.7 Amino acid analysis of anchovy protein isolate (API)

Amino acid analysis of API was determined following the method developed by Turkiewicz et al. [26]. The API (0.2 g) was dissolved in 0.5 mL of methanol and water (1:1, v/v). Then the mixture was sonicated for 15 min and centrifuged at 19,000×*g* for 10 min at 4 °C. The resulting supernatant was derivatized with the following: 350 μL of borate derivative buffer (0.2 M sodium borate, pH 8.8, with 5 mM calcium disodium EDTA), 50 μL of amino acid standards or sample extracts, and 100 μL of 10 mM aminoquinolyl-N-hydroxysuccinimidyl carbamate (AQC) reagent in elution solvents (MeOH (7:3, v/v) (A) and 0.1 % formic acid (B)). The mixture was placed into a propylene vial and vortexed (Thermo Scientific H6KT18137, Korea) for a few seconds. After 1 min at room temperature, the vials were placed into the ThermoMixer (Eppendorf ThermoMixer® F2.0, Germany) and heated at 55 °C for 10 min. After that, the samples were analysed using ultra performance liquid chromatography (UPLC) with a photodiode array (PDA) (Water Acquity DT-0200, USA). Separation of amino acids was carried out using an AccQ Tag Ultra BEH column (2.1 × 100 mm, 1.7 μm). The injection volume was 3 μL, and the elution was carried out at a flow rate of 0.50 mL/min. The mobile phase consisted of solvent A (50 mL solution: acetonitrile, formic acid and 5 mM ammonium acetate (10:6:84, v/v/v) in 950 mL water) and solvent B (acetonitrile and formic acid; 99.9:0.1, v/v). Amino acid PDA spectra were measured at a wavelength of 260 nm. Retention times and spectra were compared with amino acid standards. Quantification was achieved by injecting solutions with 20.00–100.00 mg/L of amino acid standards. Amino acid composition was calculated based on the peak area relative to the standard. Amino acids are expressed in % of amino acids in a protein sample.

### 2.8 Proximate analysis of anchovy flour (AF), defatted anchovy flour (DAF), anchovy protein isolate (API), and patty meat analogue (PMA)

The proximate analysis included moisture content (gravimetric method), ash (dry ashing method), protein (Kjeldahl method), lipid (Soxhlet method), and carbohydrates (by difference method). The proximate analysis was carried out using the Association of Official Analytical Chemist (AOAC) method [27].

### 2.9 Total energy of patty meat analogue (PMA)

The total energy calculation for the PMA was obtained from equation (3) [28].

(3) $$Total\;energy\;\left(kcal/g\right)=\left(protein\;content\;x\;4\;kcal/g\right)+\left(lipid\;content\;x\;9\;kcal/g\right)+\left(carbohydrate\;content\;x\;4\;kcal/g\right)$$

### 2.10 Analysis of the physical properties of patty meat analogue (PMA)

#### 2.10.1 Colour

Colour analysis was performed using a colorimeter (NR200, China) with values of lightness (L*) ranging from 0 to 100 representing white-black, redness (a*) ranging from 0 to 100 representing green-red, and yellowness (b*) ranging from 0 to 100 representing yellow-blue.

#### 2.10.2 Cooking loss

The cooking loss analysis of PMA followed the method of Zhang et al. [29] with modifications to cooking and cooling temperatures. Measurement of cooking loss was carried out using the water bath method. The PMA was cut into 1 × 1 × 1 cm (1 cm^3^) dimensions and weighed. Afterwards, the sample was heated in a water bath at 85 °C for 30 min. The PMA was removed and placed in cold water at 4 °C. Then, the sample was removed and gently patted with a tissue to dry the sample. Cooking loss was measured using the following equation (4).

(4) $$Cooking\;loss\;\left(\%\right)=\left(\frac{sample\;weight\;before\;cooking\;\left(g\right)-sample\;weight\;after\;cooking\;\left(g\right)}{sample\;weight\;before\;cooking\;\left(g\right)}\right)x\;100\%$$

#### 2.10.3 Texture

The PMA dimensions of 2 × 2 × 2 cm were measured for texture using a texture analyzer (TA.XT plus, Stable Micro Systems, UK) at a speed of 1 mm/s, a depth of 2 cm, using a P/35 probe (35 mm) in diameter. The parameters measured were hardness, springiness, cohesiveness, chewiness, and resilience. Hardness is the maximum force during the first compression or a measure of how hard a product is, expressed in grams (g). Springiness shows how much the product returns to its original shape after the first compression (dimensionless). Cohesiveness is the ratio of the area of the positive force of the second compression to the area of the positive force of the first compression, dimensionless. Chewiness results from hardness times cohesiveness times springiness, expressed in g. Resilience indicates how a sample recovers from deformation, including speed and strength. Resilience is defined as the ratio of the area before the deformation target to the area after the deformation target when the first compression (dimensionless).

### 2.11 Sensory analysis of patty meat analogue (PMA)

Sensory analysis was performed according to Meilgaard et al. [30]. Sensory evaluation was carried out using a hedonic test using 35 untrained panellists (10 men and 25 women) aged 19–23 years. The panellists were students of the Faculty of Biotechnology, Atma Jaya Catholic University of Indonesia. This study was ethically approved by Ethics Development Center, Atma Jaya Catholic University of Indonesia (0007P/III/PPPE.PM.10.05/08/2023). Written informed consent for this study was obtained from the panellists. The meat analogue was fried at 175 °C for 2 min before the sensory test. Each panellist was given four meat analogue samples with different formulas—every sample was named with a random three-digit code. Panellists assessed colour, aroma, taste, texture, aftertaste, and overall preference. The test scale ranges from 1 to 7, namely 1: dislike very much, 2: dislike moderately, 3: dislike slightly, 4: neither like nor dislike, 5: like slightly, 6: like moderately, and 7: like very much.

### 2.12 Statistical analysis

The data obtained were the result of three treatment repetitions. Statistical analysis was performed with SPSS 25.0 series. The normality of the data was tested from the data obtained using the Kolmogorov Smirnov test. If the data were not normally distributed, the Kruskal Wallis test could be used. Then, this could be proceeded with another test using Mann Whitney. If the data obtained were normally distributed, it could proceed to the analysis of variation (ANOVA). Then, a different test was performed using Duncan's test. Statistical tests were conducted at a significance level of 5 %.

## 3 Results and discussions

### 3.1 Yield and protein recovery of anchovy protein isolate (API)

The resulting API yield was 24.33 ± 0.02 % wb from fresh anchovies, higher than that of protein isolate from lantern fish (19.15–22.43 %) [13]; catfish (13.20–23.96 %) [15]; yellowfin tuna roe (11.6–14.1 %) [31]; and slipper cupped oysters (10.95 %) [32]. However, this API value was lower than in previous studies of 26.39 % [14]; sardine by-product (39.46 %) [33]; and dark muscle catfish (34.23 %) [34]. API yield results were lower than previous anchovy studies, perhaps because the raw anchovy ingredients contained more non-muscle protein, such as stroma which could not be extracted using alkaline, acidic, water, and salt solutions [35]. Therefore it is necessary to remove it while making protein isolates. Based on research conducted by Tan et al. [36], the yield of API in this study can already be commercialized because it has exceeded 20 %. API had a protein recovery of 36.62 ± 0.02 % wb, higher than that of protein isolates from catfish (12.33–22.04 %) [15]; tilapia frames (16.54–19.19 %) [37]; and bigeye snapper head (6–12 %) [38]. The yield and protein recovery of protein isolates are affected by the fish species, the concentration of water-soluble sarcoplasmic protein, centrifugation speed, time, and temperature during the protein isolation process [39]. The higher the yield and protein recovery, the more efficient and successful the protein isolation process is because the protein contained in the final product does not differ much.

### 3.2 Amino acid of anchovy protein isolate (API)

Amino acid composition is important to determine the quality of protein. The amino acid profile of the API is presented in Table 1. API had a high content of essential amino acids (EAA) and nonessential (NEAA). API was high in the amino acid glutamic acid (81.72 mg/g protein), leucine (71.29 mg/g protein), and phenylalanine (71.17 mg/g protein). Abdollahi & Undeland [11]; Kakko et al. [16]; and Zhong et al. [40] reported that protein isolates from silver carp, salmon, cod, and herring had the highest glutamic acid content. The EAA content of API was 451.75 mg/g protein. The EAA ratio of API was higher than NEAA. This is because protein isolation using the pH shift method can increase the EAA value compared to the raw material [16]. Studies by Abdollahi & Undeland [11]; Kakko et al. [16]; and Zhong et al. [40] also reported high EAA content in protein isolates from silver carp, salmon, cod, and herring. The EAA level of API was 72.73 %, above the amino acid requirements of the Food and Agriculture Organization of the United Nations/World Health Organization/United Nations University (FAO/WHO/UNU) [41]. EAAs that met FAO/WHO/UNU standards include histidine, threonine, valine, isoleucine, leucine, phenylalanine, tyrosine, and tryptophan. While the EAAs were under the FAO/WHO/UNU standards, namely methionine, cystine, and lysine. API can be an additive in developing high-protein food products for adults and children who require high EAA content.

**Table 1:** Amino acids of anchovy protein isolate (API) compared to FAO/WHO/UNU standards.

Amino acids	Total (mg/g protein)	FAO/WHO/UNU standards for adults (≥18 years) (mg/g protein)^a^	FAO/WHO/UNU standards for children (1–10 years) (mg/g protein)^a^
Essential amino acids (EAA)			
Histidine (His)	27.21 ± 0.08	15	16–18
Threonine (Thr)	50.19 ± 0.21	23	25–27
Valine (Val)	51.86 ± 0.29	39	40–41
Methionine (Met)	6.06 ± 0.00	22	23–25
Isoleucine (Ile)	50.68 ± 0.18	30	30–31
Leucine (Leu)	71.29 ± 0.33	59	61–63
Phenylalanine (Phe)	71.17 ± 0.39	38	41–46
Tyrosine (Tyr)	54.54 ± 0.23	38	41–46
Tryptophan (Trp)	12.42 ± 0.06	6.0	6.6–7
Cystine (Cys)	15.49 ± 0.01	22	23–25
Lysine (Lys)	40.84 ± 0.18	45	48–52
Non essential amino acids (NEAA)			
Arginine (Arg)	51.14 ± 0.25	–	–
Serine (Ser)	40.62 ± 0.17	–	–
Glutamic acid (Glu)	81.72 ± 0.35	–	–
Alanine (Ala)	35.88 ± 0.16	–	–
Glycine (Gly)	37.40 ± 0.14	–	–
Aspartic acid (Asp)	53.34 ± 0.20	–	–
Proline (Pro)	25.18 ± 0.10	–	–
Total amino acid (TAA)	777.03		
Total essential amino acids (TEAA)	451.75		
Total non essential amino acids (TNEAA)	325.28		

Data shown are means±standard deviations from two repetitions (n = 2).

a Recommended amino acid scoring pattern for adults (≥18 years) and children (1–10 years) by the FAO/WHO/UNU [41].

### 3.3 The proximate composition of fresh anchovy (FA), anchovy flour (AF), defatted anchovy flour (DAF), and anchovy protein isolate (API)

The proximate composition of API was significantly different from FA, AF, and DAF (*p* < 0.05) (Table 2). If viewed from the standards made by Codex [42], the moisture content of API (8.36 ± 0.33 % db) was still sufficient because it did not exceed 10 %. The API had a high ash content (6.13 ± 0.14 % db) because the anchovy used had a high ash content of 13.53 % db. In addition, anchovies are rich in minerals such as iron (58.16 %), calcium (5.04 %), and zinc (0.06 %) [43]. The ash content of API met the Codex standard for protein isolates, which does not exceed 8 % [42]. The protein content of FA was significantly different from AF and decreased after the flouring process (*p* < 0.05). This is possible because prolonged exposure to high-temperature treatment on drying can cause proteins to denature, thereby reducing protein content [44]. The API had a high protein content (87.23 ± 0.54 %), so it could be used as an alternative to soy protein isolate, which is still imported. In addition, API can also be used as a source of protein-rich and easily digestible ingredients in meat analogue formulations.

**Table 2:** The proximate composition of fresh anchovy (FA), anchovy flour (AF), defatted anchovy flour (DAF), and anchovy protein isolate (API).

Properties	FA	AF	DAF	API	Standard values for protein isolate^b^
Moisture (%db)	434.76 ± 0.8^a^	6.26 ± 0.10^d^	11.74 ± 0.14^b^	8.36 ± 0.33^c^	≤10
Ash (%db)	13.53 ± 0.08^a^	13.19 ± 0.06^ab^	10.08 ± 0.07^c^	6.13 ± 0.14^d^	≤8
Protein (%db)	77.89 ± 0.11^b^	71.19 ± 0.23^d^	73.90 ± 0.13^c^	87.23 ± 0.54^a^	≥90
Lipid (%db)	2.91 ± 0.04^c^	7.81 ± 0.10^a^	4.55 ± 0.04^b^	3.99 ± 0.06^b^	
Carbohydrate (%db)	5.67 ± 0.15^a^	1.92 ± 0.03^c^	0.96 ± 0.01^d^	2.52 ± 0.58^b^	

Data shown are means ± standard deviations from three repetitions (n = 3).

Different letters in the same row indicate significant differences (*p* < 0.05).

b Standard values for protein isolate by Codex [42].

### 3.4 The physical properties of patty meat analogue (PMA)

The results of the physical properties of PMA are shown in Table 3. The lightness (L*) values in F1, F2, and F3 were significantly different from F0 (*p* < 0.05). The L* value of the PMA increases along with increased API concentration. That is because the presence of fish tissue affects the lightness level of fish protein isolate. In addition, the fat content in the API can cause more light reflection, thereby contributing to the lightness level of PMA [45]. The redness (a*) values of F1, F2, and F3 were not significantly different from F0 (*p* > 0.05). However, F1 significantly differed in a* value from F3 (*p* < 0.05). Commercial meat analogues use soy, pea protein, and beets to create a meaty red colour. Legumes such as soybeans contain red symbiotic haemoglobin (leghemoglobin) [46]. The PMA a* value is influenced by adding API, tomatoes, and red yeast rice powder in the PMA preparation. The reddish colour in API is caused by the co-precipitation of heme proteins during protein isolation. Tomatoes have a red pigment, namely lycopene. Red yeast rice powder has a red pigment produced by the mould *Monascus* sp. The yellowness (b*) values of F1, F2, and F3 were also not significantly different from F0 (*p* > 0.05). However, the value of b* for F1 was significantly different from F2 and F3 (*p* < 0.05). The API had a reddish-purple to yellow colour, thus contributing to the yellow colour of PMA. The lipid content in API also contributes to the yellow colour of PMA products [14]. De Marchi et al. [46] reported that the a* and b* values of plant-based burgers were higher than meat-based burgers, but the two burgers had the same L* value. Adding API with different concentrations only slightly increased the a* and b* values of PMA. Interestingly, the resulting PMA has a red colour similar to red meat.

**Table 3:** The physical properties of Patty Meat Analogue (PMA).

Properties	F0	F1	F2	F3
Colour				
Lightness (L*)	38.95 ± 0.34^c^	40.48 ± 0.69^b^	42.02 ± 1.31^ab^	41.24 ± 0.28^b^
Redness (a*)	12.28 ± 0.23^ab^	11.38 ± 0.96^c^	12.28 ± 0.42^ab^	12.99 ± 0.71^a^
Yellowness (b*)	6.27 ± 0.15^ab^	4.98 ± 0.79^b^	6.68 ± 0.20^a^	6.89 ± 1.18^a^
Cooking loss (%)	22.02 ± 1.24^a^	18.06 ± 1.03^b^	14.50 ± 1.56^c^	12.79 ± 0.34^c^
Texture profile				
Hardness (g)	780.67 ± 7.57^d^	911.17 ± 5.48^c^	1,133.67 ± 12.05^b^	1,447.17 ± 13.29^a^
Springiness (dimensionless)	0.62 ± 0.03^d^	0.72 ± 0.03^c^	0.81 ± 0.02^b^	0.92 ± 0.02^a^
Cohesiveness (dimensionless)	0.84 ± 0.01^d^	0.95 ± 0.01^c^	1.12 ± 0.01^b^	1.22 ± 0.02^a^
Chewiness (g)	405.66 ± 18.70^d^	619.29 ± 29.90^c^	1,023.14 ± 32.37^b^	1,631.40 ± 50.52^a^
Resilience (dimensionless)	0.45 ± 0.05^d^	0.63 ± 0.03^c^	0.88 ± 0.00^b^	1.05 ± 0.05^a^

Data shown are means ± standard deviations from three repetitions (n = 3).

Different letters in the same row indicate significant differences (*p* < 0.05).

Formulations: F0 (0 % API); F1 (4 % API); F2 (8 % API); F3 (12 % API).

The cooking loss values of F2 and F3 were lower and significantly different from F0 and F1 (*p* < 0.05). Cooking loss of F3 had the lowest value (12.79 ± 0.34 %) compared to other treatments, indicating that API as a binder agent plays a role in water and lipid binding capacity. Canti et al. [19] reported that adding non-meat protein, namely jack bean protein isolate, can be used as a binding agent in chicken nuggets because it can reduce cooking loss and improve texture. That was also reported by Baugreet et al. [47] where pea protein isolate can reduce cooking loss in restructured beef steak. Cooking loss in plant-based burgers is lower than in meat-based burgers due to the high fibre content in plant-based burgers [46]. The API can increase the meat mixture's solubility and reduce water expulsion during cooking.

The textural properties of PMA are shown in Table 3. There were significant differences (*p* < 0.05) for hardness, springiness, cohesiveness, chewiness, and resilience between all samples analysed. All texture properties of PMA increased with a higher concentration of API. Protein isolates can form gels and retain water in a 3-dimensional (3D) structure. The protein isolate gel structure is formed due to hydrophobic interactions, hydrogen bonds, and disulfide bonds [48]. Gels are formed by unfolding and then forming 3D bonds randomly, which are vital to produce a compact texture [49]. Yong et al. [50] reported that adding soy protein isolate could increase the hardness, springiness, cohesiveness, and chewiness of the meat emulsion. Higher pea protein isolate could increase the mechanical strength (hardness, chewiness, and springiness) of fibrous meat analogues [9]. According to research by Mabrouki et al. [51], the resilience value of plant-based patties with pea protein isolate was higher than that of meat patties. Shoaib et al. [52] also reported that the addition of rice protein isolate to chicken nuggets could improve texture due to a decrease in the water content of the product. Ferawati et al. [53] reported that the production of meat analogues with yellow pea protein isolate and faba bean isolate had a higher hardness value than commercial meat analogues from soybeans. The ash content of protein isolate can affect the texture of the meat analogue. Calcium ions play an essential role in forming a fibrous texture, resulting in aggregation and texture in the meat analogue. The API had a high ash content of 6.13 ± 0.14 % db, thereby contributing more ionic bonds, which can improve the texture of PMA. The total dietary fibre and fat content of the protein isolate also influence the hardness value of the meat analogue. Botella-Martínez et al. [54] reported that higher fibre content in cooked burgers made with commercial juice could increase their hardness value. Non-nitrogen components such as polysaccharides will interact with proteins to increase the product's stickiness. Another factor that affects the texture value of the meat analogue is the WHC value of the protein isolate. A low WHC value indicates more protein in its native state, resulting in increased interactions between proteins and other components, such as polysaccharides, which can improve the texture of the meat analogue [53].

### 3.5 The proximate composition of patty meat analogue (PMA)

The proximate composition of PMA is presented in Table 4. The more API that was added, the higher the protein, lipid, and total energy content of PMA. Conversely, the higher levels of addition of API resulted in reduced moisture content of PMA. Moisture analysis is an essential parameter in determining the quality of meat products. The moisture content of PMA was lower than a beef patty based on the United States Department of Agriculture (USDA) National Nutrient Database (53.9 %) [55]. The moisture contents of all PMA treatments were significantly different (*p* < 0.05). The addition of API to PMA products reduced the moisture content, similar to the study by Shoaib et al. [52], where adding pea and rice protein isolates reduced the chicken nuggets' moisture content. Bakhsh et al. [25] also reported that the moisture content of plant-based meat analogue with the addition of soy protein isolate was lower than beef meat's. That is because protein isolate has a high water holding capacity (WHC), so more water is needed to hydrate the product.

**Table 4:** The proximate composition of Patty Meat Analogue (PMA).

Properties	F0	F1	F2	F3	SNI for meat burger^c^	SNI for combination meat burger^c^	USDA standard for beef patty^d^
Moisture (%wb)	54.34 ± 0.20^a^	53.33 ± 0.20^b^	52.75 ± 0.26^c^	51.74 ± 0.16^d^			53.9
Ash (%wb)	2.70 ± 0.03^c^	2.76 ± 0.01^b^	2.80 ± 0.01^a^	2.72 ± 0.01^ab^			0.9
Protein (%wb)	17.64 ± 0.50^c^	19.58 ± 0.24^b^	19.73 ± 0.17^b^	21.11 ± 0.20^a^	≥13	≥8	23
Lipid (%wb)	0.68 ± 0.01^c^	0.98 ± 0.01^b^	0.58 ± 0.01^d^	1.34 ± 0.01^a^	≤20	≤20	21.8
Carbohydrate (%wb)	24.64 ± 0.72^a^	23.35 ± 0.42^b^	24.14 ± 0.10^ab^	23.10 ± 0.04^b^			0
Total Energy (kcal/g)	175.22 ± 0.94^c^	180.59 ± 0.80^b^	180.70 ± 0.95^b^	188.84 ± 0.59^a^			295

Data shown are means ± standard deviations from three repetitions (n = 3).

Different letters in the same row indicate significant differences (*p* < 0.05).

Formulations: F0 (0 % API); F1 (4 % API); F2 (8 % API); F3 (12 % API).

c Standard of meat burgers and combination beef burgers by SNI [58].

d Standard of beef patty by USDA [55].

As seen in Table 4, the ash contents of F1 and F2 differed significantly from the control, F0 (*p* < 0.05). On the other hand, F3 had an ash content that was not significantly different from the control (*p* > 0.05). F2 had the highest ash content of 2.80 ± 0.01 % wb. The ash content of PMA (5.65–5.94 % db) was higher than that of the meat analogues of pea protein isolate and soy protein isolate (3.86–4.77 % db) [20]. Imitation burgers contain significantly higher levels of minerals such as iron, calcium, and potassium than beef burgers [56]. De Marchi et al. [46] also reported that the ash content of plant-based burgers was higher than meat-based burgers. The PMA in this study also had a higher ash content than the USDA standard for beef patties (0.9 %) [55]. The ash content of meat analogue is influenced by the proximate content of the materials used. The API had a high ash content value (Table 2), so the PMA produced had a high ash content.

Protein is one of the leading nutritional characteristics in food products, especially patty products. The protein content of all PMA treatments was higher and significantly different from the control (*p* < 0.05). F3 had the highest protein content of 21.11 ± 0.20 % wb. The increase in PMA protein content was affected by API, which formed an aggregate protein network in the mixture, where API had a high protein content of 87.23 ± 0.54 % db. That is similar to a study by de Angelis et al. [20], where adding pea protein isolate and soy protein isolate increased the levels of protein meat analogues. Swing et al. [57] also reported that plant-based meat analogues protein levels from pea protein isolate; pea protein; soy protein concentrate; and cooked black beans (17.2–24 % db) were higher than that of the ground beef patty (17 % db). The protein content of all PMA treatments met the Indonesian national standard (SNI) for meat burgers (minimum 45 % meat content) and combination beef burgers (minimum 25 % meat content) with a minimum protein content of 13 % and 8 %, respectively [58]. The PMA protein content in this study also met the protein content range of commercial meat analogues of 10.17–25 % [59]. PMA in this study had protein levels (19.58–21.11 % wb), which were not much different from McDonald's beef patty (23.33 %) and ground beef patty based on the USDA National Nutrient Database (23 %) [55,60]. PMA is an alternative consumption substitute for meat so that consumers can switch to a sustainable diet.

The lipid content of all PMA treatments was significantly different from the control (*p* < 0.05). All PMA treatments met SNI for meat burgers and combination meat with less than 20 % lipid content. Swing et al. [57] reported that plant-based meat analogues of pea protein isolate; pea protein; soy protein concentrate; and cooked black beans have a lipid content of 10.58–13.1 % db. The lipid content of PMA was lower than that of commercial meat analogue products (5.56–15.93 %); ground beef patty based on the USDA National Nutrient Database (21.8 %); and McDonald's beef patty (20 %) [55,59,60]. PMA products in this study can be categorized as low fat because they have a lipid content of less than 2 % wb. Burgers meat analogues with low-fat content (total saturated and unsaturated fats) and calories provide health-related benefits such as preventing obesity, diabetes, cholesterol, and cardiovascular disease [56].

The carbohydrate content of F1 and F3 differed significantly from F0 (*p* < 0.05). However, the level of F2 carbohydrates was not significantly different from the control (*p* > 0.05). Bohrer [59] reported that the carbohydrate content of commercial meat analogues was 2.65–24.58 %. The carbohydrate content of PMA comes from API and sago flour which is added when making PMA. Sago flour has a carbohydrate content of 83.92 % [61]. Sago flour increases product consistency, while API is used as a binder in meat analogues. According to Cole et al. [56], imitation and veggie burgers contain higher carbohydrates than beef burgers because they have high levels of fibre and sugar.

The total energy of all PMA treatments was significantly different from the control (*p* < 0.05). F1 and F2 showed total energies that were not significantly different (*p* > 0.05). PMA had lower total energy than that of ground beef patty based on the USDA National Nutrient Database (295 kcal/100 g) and McDonald's beef patty (266.67 kcal/100 g) [55,60]. PMA's total energy was still in the range of commercial meat analogue products (140.85–221.24 kcal/100 g) [59]. Cole et al. [56] reported that the calorie content of imitation and veggie burgers with the same serving size was significantly lower than beef burgers. That is because beef burgers have higher total fat, saturated fat, *trans*-fat, and cholesterol than imitation and veggie burgers. In addition, the total energy value is affected by the amount of protein and carbohydrate content in the meat analogue. PMA consumption has the advantage that it can reduce excess calorie intake and have a good impact on health in the long term.

### 3.6 Sensory properties of patty meat analogue (PMA)

**Fig. 1:**
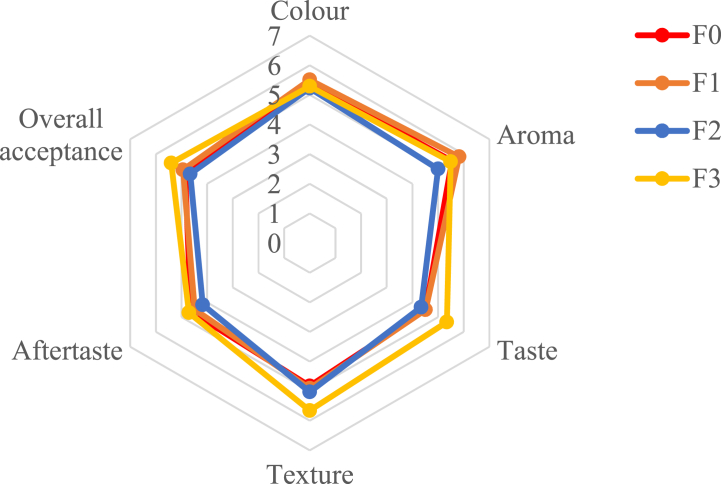
Sensory evaluation of Patty Meat Analogue (PMA), n = 35. Formulations: F0 (0 % API); F1 (4 % API); F2 (8 % API); F3 (12 % API).

The sensory characteristics of PMA are presented in Fig. 1. Based on the sensory analysis results, adding different concentrations of API did not significantly affect the colour, aroma, texture, aftertaste, nor overall preference of PMA (*p* > 0.05). Panellists liked the colour of all the PMA treatments, where the PMA was red, resembling a beef-meat patty. TVP in the PMA formulation gives a legume aroma with low intensity and a meaty aroma. Similar to the research by de Angelis et al. [20], meat analogues with pea and soy protein isolates have aromas of legumes, cereals, and sweetness, and are meat-like with low intensity. PMA in all treatments had a meat-like, legume, umami, saltiness, cereal, and sweet taste. The higher the concentration of API added, the stronger the protein unfolds to form 3D bonds randomly so that the texture is more compact, resembling real meat and is liked by the panellists. The aftertaste of PMA is due to the presence of bitterness and astringency. According to de Angelis et al. [20], plant-based protein presents bitterness and astringency caused by the presence of antinutritional factors (saponins) and phenolic compounds (tannins, catechins). API also had a taste of bitterness and dried fish caused by the lipid, ash, and bile content in whole fish and offal [14]. Even though it had an aftertaste, PMA is still preferred by panellists because of the low intensity of bitterness and astringency. The more API that was added, the more PMA was preferred by panellists on all sensory attributes (colour, aroma, taste, texture, and aftertaste). Based on sensory evaluation, F3 was the best treatment.

## 4 Conclusions

This study successfully produced an API with a high protein content of 87.23 % db demonstrating that API can be used as an alternative source of protein other than soy protein isolate. The API contained 451.75 mg of essential amino acids per g of protein, most of them met the requirement of amino acids for adults and children. The addition of API significantly increased the colour and texture of PMA and reduced the value of cooking loss in PMA. Also, the addition of API also significantly affects the content of moisture, ash, protein, lipid, carbohydrates, and the total energy of the PMA produced. All treatments at different API concentrations contained protein and lipids that met the SNI standards for meat burgers and combinations. The protein content of all treatments in PMA was also not much different from the beef patty from McDonald's and the USDA standard for the ground beef patty. Interestingly, PMA has a very low-fat content, so it has a good impact on health. All sensory attributes of all PMA treatments were not significantly different from the control, except for taste. The more API that is added, the higher the intensity of umami will produce PMA. F3 was the best treatment, with good texture, proximate composition, and a high panellist preference level.

## Funding

This research was funded by the Directorate General of Higher Education, Research, and Technology, Ministry of Education, Culture, Research, and Technology of Republic of Indonesia through the National Competitive Research Grant 2023 (Contract No. SP DIPA-023.17.1.690523/2023, 1415/LL3/AL.04/2023, 0388.7/III/LPPM.PM10.01-HD/6/2023).

## Ethical statement

This study was ethically approved by Ethics Development Center, Atma Jaya Catholic University of Indonesia (0007P/III/PPPE.PM.10.05/08/2023).

## Informed consent statement

Informed consent was obtained from all subjects involved in the study.

## Data availability statement

Data will be made available on request.

## Additional information

No additional information is available for this paper.

## CRediT authorship contribution statement

**Meda Canti:** Writing – original draft, Visualization, Validation, Supervision, Methodology, Investigation, Funding acquisition, Data curation, Conceptualization. **Juan Owen:** Methodology, Formal analysis, Data curation. **Maximilliam Febriand Putra:** Methodology, Formal analysis, Data curation. **Rory Anthony Hutagalung:** Writing – review & editing, Validation, Supervision, Resources. **Novia Utami:** Resources, Project administration.

## Declaration of competing interest

The authors declare that they have no known competing financial interests or personal relationships that could have appeared to influence the work reported in this paper.
